# Fibular strut graft for primary ankle arthrodesis in diabetic charcot neuroarthropathy patients

**DOI:** 10.1016/j.ijscr.2023.108430

**Published:** 2023-06-30

**Authors:** Andre Triadi Desnantyo, Muhammad Hanun Mahyuddin, Pandit Bagus Tri Saputra, Olga Putri Atsira

**Affiliations:** a Medical Faculty Universitas Airlangga, Surabaya, East Java 60132, Indonesia; b Department of Orthopedics & Traumatology Dr. Soetomo General Hospital/Universitas Airlangga, Surabaya, Indonesia

**Keywords:** Fibular strut graft, Diabetes mellitus, Charcot neuroartropathy, Cost-effectiveness, Ankle arthrodesis

## Abstract

**Introduction:**

Arthrodesis is an expensive procedure that is less applicable in developing countries. In this case report we reported a case of diabetic charcot neuroartropathy (CN) with primary ankle arthrodesis technique with a fibular strut graft which is considered cheaper and has a higher union rate.

**Case description:**

A 47 years old female with complaints of pain in her right ankle after falling down the stairs with foot inverted one month before admission. The patient has uncontrolled diabetes mellitus with HbA1C 7.6 % and random blood sugar check >200 mg/dl. The patient's pain score using the visual analog score (VAS) showed a value of 8. While plain film X-ray revealed bony fragmentation in the Ankle joint. Arthrodesis surgery using fibular strut graft was performed. The postoperative X-ray examination revealed two plates attached to the anterior and medial distal tibia. A total of nine wires were attached to the patient. The patient used Ankle Foot Orthosis (AFO) and was able to walk normally 3-weeks post-surgery without pain and ulcer formation.

**Discussion:**

Fibular strut graft has good cost-effectiveness, that is more suitable for use in developing countries. It also requires a simple implant that is easily applied by all orthopedists. Fibular strut graft has the advantage of having osteogenic, osteoinductive, and osteoconductive properties that can potentially improve union.

**Conclusion:**

The fibular strut graft technique can be an alternative in obtaining durable ankle fusion and functional salvaged limb with low complications.

## Highlights

• Autologue fibular strut graft has potentially improve the outcome of ankle arthrodesis in diabetic CN
• Fibular strut graft arthrodesis can be a therapeutic option in developing countries because its more cheaper.
• Poor compliance of diabetic patient can result to lower success rate of fibular strut graft technique

## 1 Introduction

Charcot Neuroarthropathy (CN) is a systemic disease that causes weakness of the musculoskeletal system, especially in the ankles and foot region. Charcot Neuropathy is the most common cause of subluxations, dislocations, deformities, and ulcers in patients with diabetes mellitus (DM) [1]. DM patients with CN are associated with higher mortality and the risk of amputation up to 40 times higher compared to DM patients without CN [2]. The prevalence of CN in patients with Diabetes Mellitus (DM) reaches 7.5 % and may be higher due to underdiagnosis [3]. Although this number seems small, considering the prevalence of DM in 2014 is estimated at 422 million people and continues to increase rapidly, CN becomes a serious problem especially in low-moderate income countries (LMIC) [1].

Management of CN patients includes non-surgery and surgery. Out of various non-surgical treatments, of which Total Contact Cast (TCC) is the gold standard treatment in acute Charcot neuroarthropathy [4]. Patients with exostosis, joint instability, pain with recurrent misalignment and scarring, fractures, osteomyelitis, and inability to ambulate should undergo surgery [5]. Arthrodesis surgery therapy is a procedure to save limbs to avoid amputation. Ankle arthrodesis aims to maintain the patient's productivity and quality of life in CN patients.

However, due to poor vascularity and osteopenia in CN patients, arthrodesis potentially has low union rate and some complications [6]. In addition, arthrodesis is a complex procedure that requires high cost. Thus, ankle arthrodesis procedure with low costs and good outcome critically important to preserve diabetic CN patient productivity and quality of live, especially in LMIC.

This case report presented the use of the primary ankle arthrodesis technique with fibular strut graft for a female with diabetic CN patient, in line with the surgical case report (SCARE) guidelines [7].

## 2 Case description

A 47-year-old female came to the hospital with complaints of pain in her right ankle after falling down the stairs in an inverted position one month before. The pain was more pronounced during walking and standing position. The patient had diabetes mellitus since 10 years ago with low treatment compliance (HbA1C 7.6 % and random blood sugar 317 mg/dl).

**Fig. 1:**
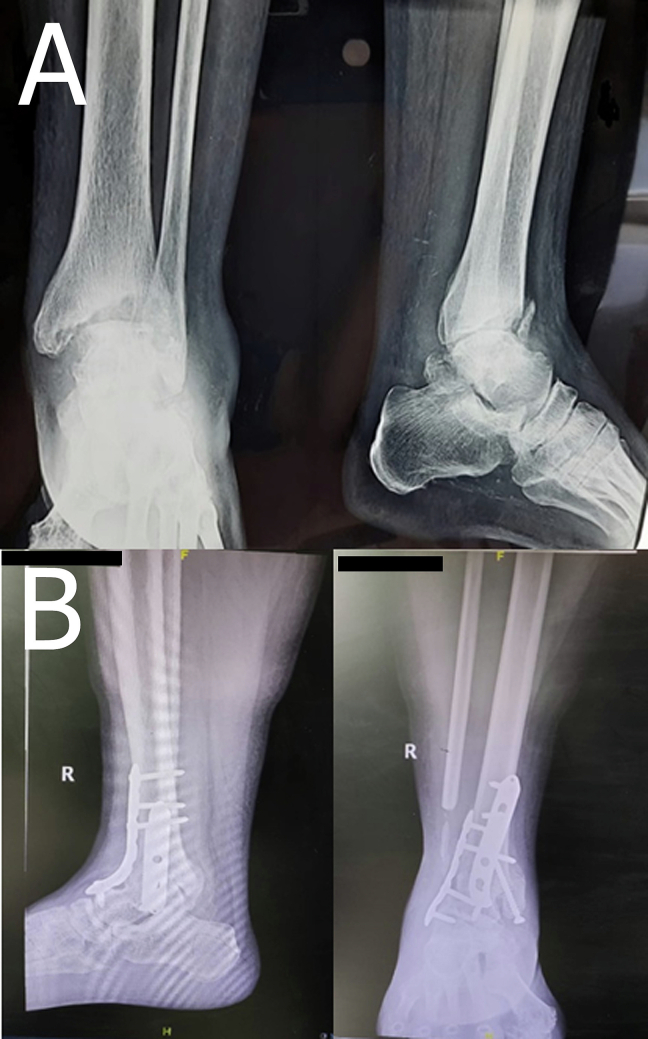
X Ray examinations. (A) Pre-operative right foot Roentgen. (B) Post-operative right foot Roentgen.

The patient was compos mentis with visual analog scale (VAS) was 8. Physical examination showed blood pressure (BP) of 126/85 mmHg, heart rate of 94×/min, temperature of 36C, respiratory rate of 20×/min, and SpO2 99 %, and body mass index (BMI) of 29.91 which falls within the obesity range. Physical examination revealed edema and tenderness of the right ankle with limited range of motion (ROM) due to ankle pain. While plain film X-ray revealed bony fragmentation in the Ankle joint (Fig. 1A). The patient refused to be amputated and underwent fibular strut graft arthrodesis.

**Fig. 2:**
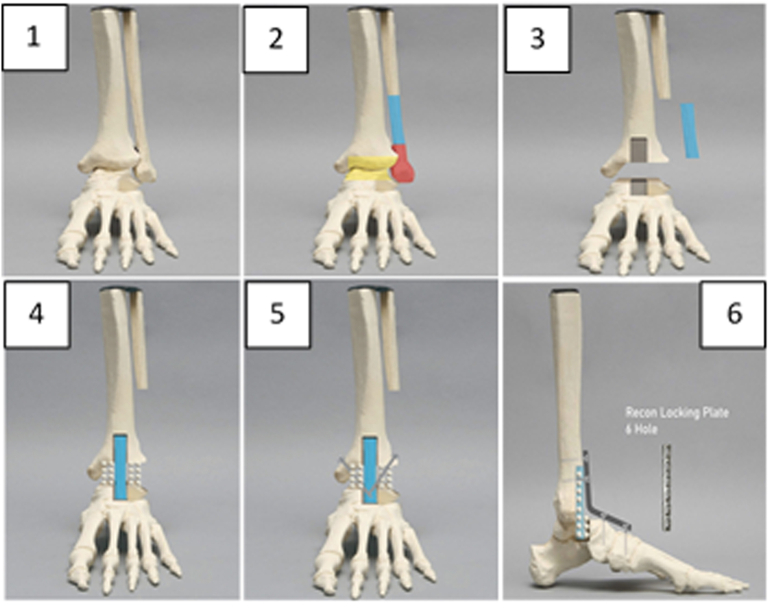
Fibular strut graft surgical technique in charcot neuroarthropathy cases. (Blue: fibular part used for fibular strut graft; yellow: debridement area in the fibular strut graft technique; red: (lateral malleolus) is not used in the fibular strut graft technique). (For interpretation of the references to color in this figure, the reader is referred to the web version of this article.)

The principle of operation technique is shown in Fig. 2. In this case, we used anterior and lateral approaches. A curved incision at the medial approach was made to access the medial osteotomy and distal fibula with a clearer view. After debridement of the necrotic tissue, we denuded the talar dome cartilage (Fig. 2.2 yellow color) to create a gutter tunneling at the anterior surface of the distal tibia and a hole at the center of talar dome to prepare the implantation of fibular strut graft. After measuring the precise size of fibular graft, we implanted fibular graft through gutter side of tibia and the hole of talus (Fig. 2.3). Autologous bone graft was added between tibiotalar surrounding fibular strut graft. (Fig. 2.4 and 2.5). We fixated the fibular strut graft with a reconstruction locking plate or small locking compression plate (adjustable bend) with nine cannulated screws for augmentation of stabilization (Fig. 2.6).

**Fig. 3:**
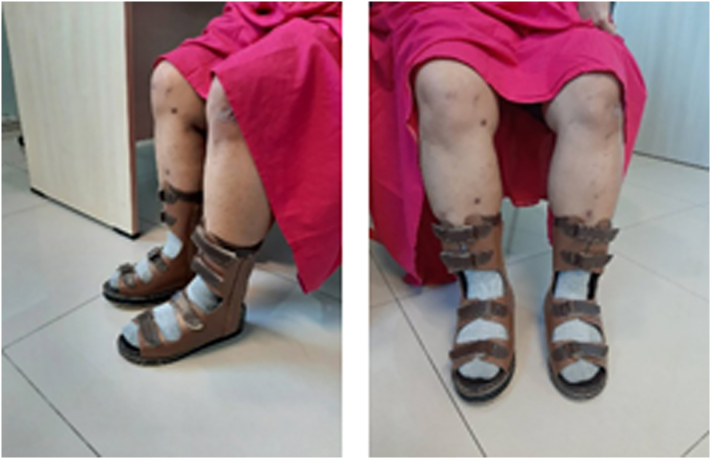
Ankle foot orthosis (AFO) was applied to the patient.

The postoperative X-ray examination revealed two plates attached to the anterior and medial distal tibia. A total of nine screws were attached to the patient (Fig. 1B). Blood glucose was strictly controlled to avoid any complications. Post-surgery, the patient was also required to wear Ankle Foot Orthosis (AFO) for 3–6 Weeks (Fig. 3) to stabilize the ankle joint. The patient was able to walk normally three weeks' post-surgery. The patient had removed the AFO at week six on the doctor's advice.

During the post-operative period, we were focusing on healing the stitches and controlling blood sugar. After a month, the pain was drastically reduced and the stitches had started to dry. In the following month, the removal of the AFO was planned. The patient was only able to receive follow-up care after six months because the patient was out of town. There was no complaint of pain during walking, infection, or ulcers in six months' follow-up.

## 3 Discussion

Arthrodesis is one of the most frequently used ways to save the CN ankle from severe joint instability and amputation. The condition of the CN ankle in DM patients is associated with severe deformity, bone osteopenia, unhealthy soft tissue and delayed healing processes making arthrodesis of the CN ankle have a high number of complications [8]. Arthrodesis failure occurs in 10–20 % of cases and increases to 62 % in high-risk cases such as the ankle [9]. The advantage and disadvantage of conservative therapy for our patients are shown in Table 1.

**Table 1:** Advantage and disadvantage surgical and non-surgical methods for treatment of charcot foot [[22], [23], [24]].

Surgical			Non-Surgical	
Advantage	Disadvantage	Indication	Advantage	Disadvantage
Able to reposition the foot optimally	Invasive action	Exostosis	Less expensive	Possibility of arch collapse
High bone fusion rate	High complication rate (36 %)	Severe instability with pain	Non-invasive	Progression of bony prominence
Reduce pain	High amputation rate (5 %)	Inability to ambulate	Effective in the acute stage of CN	Bump-related plantar pain
Reduce ulcer formation		Osteomyelitis		High ulcer occurrences

CN: charcot neuroarthropathy.

Bone grafts or bone morphogenic proteins (BMP) for ankle arthrodesis is often used in difficult cases such as revision arthrodesis, high risk of nonunion and severe deformities including ankle CN. Although BMP as an alternative to bone graft has shown satisfactory results, the emergence of BMP reports its association to cancer and the issue of very high prices have made surgeons, especially in developing countries, reconsider the decision of using BMP [10]. In this case we used autologous fibular strut graft because it is stronger and can preserve the length of the lower limb. In addition, the fibular strut graft also makes it possible to apply a simple implant for osteosynthesis. Despite the various developments of bone graft analog, autologous bone graft remains the gold standard to fill none defect in orthopedic surgery. Our procedure requires a single surgery for harvesting patient bone graft and performing ankle arthrodesis.

Fibular strut graft has a more effective cost-effectiveness and technically does not require special tools or requirements. The use of a fibular strut graft is considered more suitable in developing countries because of its cost-effectiveness superiority to arthrodesis [11]. Comparative trials conducted on patients with osteonecrosis of the femoral head who received total hip arthroplasty (THA) and free vascular fibular grafting (FVFG) surgery show that the cost of THA tends to be larger (+$5933) than FVFG and cost-effectiveness of FVFG is more effective than THA [12]. We have not found study that directly compare the cost-effective use bone graft in arthrodesis. In our case, the fibular strut graft only needed a cost mean of six million rupiah ($450) while conventional procedure required 12–21 million rupiah in our institution. The large differences probably due to the fibular strut graft doesn't need any additional implant. The mean cost of arthrodesis an amputation in CN patients is $13,511 and $25,090, respectively [13].

Fibular strut grafts itself naturally possess strong biomechanical properties and minimize implant-related complications [8]. Theoretically, the use of a vascular fibular strut graft has an advantage because it has osteogenic, osteoinductive, and osteoconductive properties which have the potential to increase union, especially in diabetic patients [9,14]. The use of fibular strut graft in arthrodesis results in resistance to all planes of motion and results in good bone fusion, including in cases of severe osteopenia [8,15]. Various reports of ankle arthrodesis show satisfactory results with fibular grafts [8,[14], [15], [16], [17]], two of them using fibular strut graft on revisional arthrodesis [8,14], fibular strut graft [8,14,16], and using transfibular strut graft [17]. The similarities, difference, and outcome from the report were concluded in Table 2.

**Table 2:** The different and similarities from another fibular strut graft technique.

No	Author	Surgical technique	Follow up and outcome	Similarities	Difference
1.	Jeong, S.T. et al. [8]	Intramedullary nonvascularized fibular graft with external fixation	Partial weight bearing at 12 weeks, removal external fixator at 3 months, short leg cast at 3 months, complete union at 6 months, at 33 months after surgery showed excellent consolidation without progression of the deformity and a well-maintained hind foot alignment	Using fibular strut graft that harvested from the patient	Placing the fibular strut graft intramedullary, used in revised patient that has implant failure due to CN, using external fixation
2.	Monaco et al. [14]	**Patient 1:** intramedullary fibular graft with external fixation	**Patient 1:** at 1 year postoperatively, the patient was ambulating with an ankle foot orthotic brace and is ulcer free with a plantar grade foot **Patient 2:** at her 4-month follow-up visit was back into regular shoe gear with minimal pain.	**Patient 1:** using fibular strut graft that harvested from the patient **Patient 2:** using fibular strut graft that harvested from the patient, Placing fibular graft as inlay strut graft	**Patient 1:** placing fibular strut graft intramedullary, using external fixation, used in revised patient that has implant failure due to CN **Patient 2:** used in revised patient with osteomyelitis due to implant after osteoarthritis surgery
3.	Ebraheim, et al. [15]	Intramedullary fibular graft with external fixation	The average follow-up was 28 months (range, 24–31 months). All the patients had successful tibiotalocalcaneal fusion and were satisfied with the results. None of the patients had mal-union or non-union. The average time between surgery and achieving fusion was 4 months (range, 3–6 months)	Using fibular strut graft	Placing fibular strut graft intramedullary, All patients were ankle fracture patients
4.	Shah et al. [16]	Intramedullary fibular graft	The mean follow-up time was 9.1 (range 9 to 18) months. Of the 16 patients, 13 (81.2 %) achieved union as confirmed by CT or radiographs. Visual analog scale scores at the final follow-up score improved from 6.9 to 1.2	Using fibular strut graft	Placing fibular strut graft intramedullary, The subject were non CN patients
5	Sung et al. [17]	Fibular onlay strut graft	–	Using fibular strut graft that harvested from the patient, Using internal fixation	Placing fibular strut graft as onlay strut graft

CT: computed topography; CN: charcot neuroarthropathy.

In our case, we used fibular strut graft placed at the anterior gutter cortex of distal tibia and center of talar dome fulfilled with bone auto graft. Shah et al. showed that three from 16 patients experienced nonunion and implant failure [16]. Another study reported 0 % of nonunion rate and amputation rate in 12 patients who underwent free vascularized fibula graft for ankle arthrodesis salvage [18]. In our case, patient could walk after three weeks postoperatively and there was no ulcer formation, as well as complaint of pain.

Technically in our technique required double incision, anterior and lateral approach (except for an additional medial approach to reach the medial malleolus). The lateral approach that we used to harvest the fibula is a classic lateral approach in arthrodesis to make it less invasive and thus lower morbidity [9,14]. We make an incision in the ankle area to do an anterior approach and a lateral approach, besides that we can also add an incision to the medial part of the ankle. Post-operative infection in CN, mostly caused by delayed wound healing, is one of the most common causes of failure in arthrodesis procedure [6]. The using of anterior approach for arthrodesis because it is more effective and does not require a new incision to speed up wound healing time and reduce the risk of infection [19]. The anterior approach is often used in classic arthrodesis for the debridement of damaged tissue and cartilage. In addition, this approach is an easier, simpler, and less resource-intensive placement of the fibular graft than the guided plantar approach [19]. An anterior approach by placing a fibular strut graft on the perforated tibia, talus, and calcaneus allows the fibular strut to have a mechanical supporting role and enhances union due to the extensive exposure of the bony surface [20].

Bone debridement is performed to remove unhealthy bone that possible disturb the realignment process [21]. Bone debridement may create a wide space between the tibiotalar joints which causes leg length discrepancy. The use of inlay fibular strut graft can maintain leg length because it can be adjusted according to the needs after bone debridement is performed.

The decision of the fixation type on the fibular strut graft depends on the surgeon's considerations based on the technique used [9,14]. In this case, we used locking plates and screws because they have good abilities and are suitable for joint immobilization and fibular grafts. The AFO is required to stabilize the ankle joint after arthrodesis procedure. In addition, the use of fibular strut graft, tight control of blood glucose is required to minimize the risk of complication following ankle arthrodesis. Although short term follow-up of our patient showed good outcome, long term follow-up is required to be evaluated.

## 4 Conclusion

Ankle arthrodesis with autologue fibular strut graft in diabetic CN is a feasible and cost-effective procedure with good short term outcome. It is a potentially recommended procedure in low-moderate income countries; however, the long-term outcome is needed to be evaluated.

## Ethical approval

Ethical approval is exempt/waived at Doctor Soetomo General Hospital because in this case reports we did not included the identity of the patient.

## Funding

This case report did not receive any specific grant from funding agencies in the public, commercial, or not-for-profit sectors.

## Declaration of competing interest

The authors declare that there is no conflict of interests regarding the publication of this paper.
